# Non-clinical assessment of lubrication and free radical scavenging of an innovative non-animal carboxymethyl chitosan biomaterial for viscosupplementation: An *in-vitro* and *ex-vivo* study

**DOI:** 10.1371/journal.pone.0256770

**Published:** 2021-10-11

**Authors:** Jean-Michel Vandeweerd, Bernado Innocenti, Guillem Rocasalbas, Sandrine Emilia Gautier, Pierre Douette, Laurence Hermitte, Fanny Hontoir, Mickael Chausson

**Affiliations:** 1 OASIS, Integrated Veterinary Research Unit, Namur Research Institute of Life Sciences (NARILIS), Namur University, Namur, Belgium; 2 BEAMS Department, Université Libre de Bruxelles, Brussels, Belgium; 3 KiOmed Pharma, Herstal, Belgium; University of South Carolina, UNITED STATES

## Abstract

**Objective:**

Lubrication and free radical scavenging are key features of biomaterials used for viscosupplementation (VS) of joints affected by osteoarthritis (OA). The objective of this study was to describe the non-clinical performance characterization of KiOmedine^®^ CM-Chitosan, a non-animal carboxymethyl chitosan, in order to assess its intended action in VS and to compare it to existing viscosupplements based on crosslinked hyaluronan (HA) formulations.

**Method:**

The lubrication capacity of the tested viscosupplements (VS) was evaluated *in-vitro* and *ex-vivo*. *In-vitro*, the coefficient of friction (COF) was measured using a novel tribological system. Meanwhile, an *ex-vivo* biomechanical model in ovine hindlimbs was developed to assess the recovery of join mobility after an intra-articular (IA) injection. Free radical scavenging capacity of HA and KiOmedine^®^ CM-Chitosan formulations was evaluated using the Trolox Equivalent Antioxidant Capacity (TEAC) assay.

**Results:**

In the *in-vitro* tribological model, KiOmedine^®^ CM-Chitosan showed high lubrication capacity with a significant COF reduction than crosslinked HA formulations. In the *ex-vivo* model, the lubrication effect of KiOmedine^®^ CM-Chitosan following an IA injection in the injured knee was proven again by a COF reduction. The recovery of joint motion was optimal with an IA injection of 3 ml of KiOmedine^®^ CM-Chitosan, which was significantly better than the crosslinked HA formulation at the same volume. In the *in-vitro* TEAC assay, KiOmedine^®^ CM-Chitosan showed a significantly higher free radical scavenging capacity than HA formulations.

**Conclusion:**

Overall, the results provide a first insight into the mechanism of action in terms of lubrication and free radical scavenging for the use of KiOmedine^®^ CM-Chitosan as a VS treatment of OA. KiOmedine^®^ CM-Chitosan demonstrated a higher capacity to scavenge free radicals, and it showed a higher recovery of mobility after a knee lesion than crosslinked HA formulations. This difference could be explained by the difference in chemical structure between KiOmedine^®^ CM-Chitosan and HA and their formulations.

## Introduction

Osteoarthritis (OA) is a chronic degenerative disease that affects the synovial joint. It is characterized by the loss of hyaline cartilage. OA is now suggested to affect the entire joint, causing cartilage degradation, synovial inflammation, subchondral bone changes, and osteophyte formation [1].

OA is the most frequent musculoskeletal disease and it affects millions of people worldwide. In addition to the burden of patients suffering from pain and disability, OA is a major concern from both an economic and social point of view. Although aging is the most common etiological factor of OA, other predisposing factors include genetics, gender, trauma, and obesity [2, 3].

The damaged cartilage is not in itself a source of pain in OA since it is not innervated. However, with damaged cartilage, friction between bones occurs, which in turn causes joint pain, abnormal function, and stiffness. Excessive friction is able to induce further chondrocyte death, a process that is mediated by free radicals [4], which gradually worsens the pathological status of the joint. To date, no treatment has been developed that could stop or reverse joint degradation; the treatments of OA patients mainly target the symptoms. Local treatment consists of intra-articular (IA) administration of anti-inflammatory agents and VS [5].

VS is a procedure that involves a viscous or viscoelastic fluid, such as hyaluronic acid (HA) formulations, being injected into the articular cavity to complement synovial fluid. An IA injection of VS is an effective treatment for mild to moderate knee OA, and a well-tolerated treatment of knee and other joints OA [6]. In healthy joints, the synovial fluid (SF), a non-Newtonian fluid (with a viscosity dependent on the shear rate), provides low-friction and low-wear properties to articular cartilage surfaces [7]. This lubrication capacity is linked to physicochemical and rheological properties of SF, and the ability of its components to interact between them and with the cartilage surface such as HA and lubricin [8–10]. Lubricin is a superficial zone (glycol)protein (SZP) secreted by synoviocytes but also by superficial zone chondrocytes [11], With abundant negatively charged and highly hydrated sugar groups, lubricin plays a fundamental role in lubrication at the boundary surface of cartilage due to strong repulsion via steric and hydration forces [8, 11–14]. Additionally, it has been observed that the interaction of lubricin with HA allows diminishing the shear energy by joint gliding [9, 15].

HA is the main component of SF and it plays a crucial role in joint lubrication mechanism. In OA, the reduction of the rheological properties of SF is likely due to the decrease in HA mean molecular weight and concentration. This HA degradation leads to SF losing its ability to lubricate the joint. One cause of this is the oxidative degradation of endogenous lubricating HA by free oxygen radicals [16–18]. Such free radicals are involved in the production of some biochemical factors that are associated with cartilage aging, cartilage degradation, and joint inflammation [19].

Currently, the most popular commercially available VS are made of linear or crosslinked HA formulations. Treatment with the linear HA VS occurs via multiple IA injections, while treatment with the crosslinked HA VS can be done via a single injection treatment regimen. When well managed, the crosslinking technology allows to prolong the residence time of a safe VS in the joint by creating a 3-dimensional HA network, which in turn enables the limiting of the number of injections per treatment cycle and the reduction of OA symptoms for at least 6 months [20]. To date, it is believed that it allows for single (IA) injection performance.

KiOmedine^®^ CM-Chitosan is a proprietary chitosan derivative that is obtained after extraction from the edible white mushroom Agaricus bisporus [21, 22]. In addition to its excellent safety profile [23], KiOmedine^®^ CM-Chitosan, has demonstrated lubrication capacity and a superior ability to fight against oxidative stress thanks to its free radical scavenging properties. As these properties are crucial in the symptomatic treatment of OA, a VS formulation comprising 2% KiOmedine^®^ CM-Chitosan has been developed for the treatment of painful knee OA.

Chitosan is one of the most abundant polysaccharides in nature and it can be extracted from various animal or non-animal sources [24]. Chitosan and its derivatives have been extensively investigated for many medical and cosmetic applications, including wound dressings, oral hygiene, hair care, contact lenses, drug delivery, and tissue engineering [25]. KiOmedine^®^ CM-Chitosan, carboxymethyl chitosan from fungal source, is soluble at physiological pH and it has several advantages over other polymers, such as nontoxicity, biocompatibility, immunocompatibility, bioresorption, and antioxidant (namely free radical scavenging) capacity. All of these characteristics of our KiOmedine^®^ CM-Chitosan have supported the development of the new VS. When formulated as a viscous biomaterial and injected into the joint, KiOmedine^®^ CM-Chitosan would exhibit lubrication capacity and free radical scavenging ability.

In orthopaedic research and development, to move new technologies or new products from bench to bedside, preclinical studies using translational animal models are required. The Osteoarthritis Research Society International (OARSI) has published guidelines for histological assessment of OA joints in different species in an attempt to standardize the assessment and reporting of animal studies [26]. Preclinical studies evaluating the healing of cartilage have been performed using both small and large animal models including murine, lapine, porcine, caprine, ovine, canine and equine models [27, 28]. The need to mimic the dimensions and loading experienced by an adult human joint motivates investment into large animal models of OA. The most commonly utilized large animals are pigs, goats, horses and sheeps. The importance of research on cartilage repair and the use of an ovine model were well described in a recent review; the knee of the sheep has been the most commonly used large animal model between 2013 and November 2017 (~5 years) [29].

However sheeps are not prone to spontaneous OA and surgical methods are used to induce the disease by destabilization of the joint. A recent paper reviewed in vivo studies, performed in sheep and published in the last ten years. In the 25 included studies, the most frequently performed technique is the unilateral total medial Meniscectomy (Mx) that increases changes in cartilage and subchondral bone more than the other techniques [30].

However, this review highlighted the fact that it is difficult to conclude with certainty what stage of OA is generated by each type of Mx. Since individual variations in cartilage changes are expected and this could modify outcomes such as biomechanics, and besides there is a need nowadays to reduce (3R’s) the number of animals that are used for research, we think efforts of researchers must also concentrate on the development of ex vivo models. In this study we aimed to develop and use a new ex vivo ovine model in which we would create standardized chondral defects on the axial aspect of the medial femoral condyle since it is a prevalent site of naturally occurring cartilage defects in this population of research sheep [31].

The objective of the current study was to assess the free radical scavenging effect and the lubrication capacity of KiOmedine® CM-Chitosan in dedicated *in-vitro* and *ex-vivo* models, in comparison to two commercially available VSs based on crosslinked HA (Hylan G-F 20 technology and NASHA technology).

## Materials and methods

### Viscosupplements

#### NASHA formulation (Bioventus)

This VS is composed of 2% of stabilized (i.e. crosslinked) HA particles in physiological solution. The crosslinking agent (1,4-butanediol diglycidyl ether) reacts with hydroxyl groups of the repeating disaccharide unit, joining the HA molecules in a three-dimensional network.

#### Hylan G-F 20 formulation (Genzyme)

This VS is composed of 0.8% of crosslinked HA. It is an association of Hylan A, where the fluid part (80%) is composed of HA extracted from rooster combs via formaldehyde, and Hylan B, where the viscoelastic part (20%) is composed of Hylan A crosslinked via divinyl sulfone [32].

#### KiOmedine^®^ CM-Chitosan (KiOmed Pharma)

This new VS is a viscous biomaterial that is composed of 2% (w/w) KiOmedine^®^ CM-Chitosan in phosphate buffer supplemented with 3.5% of sorbitol under isosmotic condition. KiOmedine^®^ CM-Chitosan is a polysaccharide of N-acetyl glucosamine and glucosamine both of which are modified with carboxylic functions. It is obtained by controlled derivatization of chitosan with proprietary modification [21, 22]. The minimum degree of acetylation is 40% and a degree of carboxyalkylation (N2, O3, O6 positions) is between 50% and 200% according to NMR 1H.

### Lubrication capacity

Lubrication capacity was assessed in two experimentations. An *in-vitro* tribological model was first designed to determine the impact of the VS on frictional forces between interacting surfaces mimicking the cartilage. Then, an *ex-vivo* ovine model was used to evaluate the lubrication capacity of the biomaterials on the damaged cartilage with a higher friction and the influence of the volume of material on the improvement of joint motion and friction.

#### *In-vitro* tribological characterization

The lubricating capacity of VS was determined by measuring the coefficient of friction (COF) between two hydrated polyEGPEA/HEMA (70%EGPEA+30%HEMA) discs in the presence of the sample. With regard to its physicochemical characteristics—namely elasticity, hydrophilicity, and porosity—resembling those of healthy cartilage, pHEMA-based materials have been used as an artificial substitute to cartilage in tribological studies [33–37]. This characterization was performed on a calibrated HR-2 rheometer (TA instrument) according to the following procedure: the discs (16.15 mm) were first hydrated to saturation in water at 90°C for a minimum of 14 hours and cleaned and rinsed with water for injection. The discs were then stored at 4°C in water. From there, two discs were mounted to the upper and lower geometries of the HR-2 rheometer. A volume of VS of 100 μL was then loaded on the lower disc. The upper geometry was moved down in order to obtain close contact between the two discs achieving a normal force (nF) of 5 N. Torque was recorded as a function of step time under constant normal force at room temperature for a duration of 150 seconds, in an oscillatory motion with an oscillation frequency of ~ 6 rad/s and an oscillation angle of ~ 0.05 rad. Five repetitions of a minimum of three independent samples were analysed for each product at room temperature. The Time-course torque was plotted for each repeat and the COF was calculated as the mean y-intercept of the 5 curves. Under these conditions, and taking into consideration the diameter of the disk (ø), the COF was calculated at each time point as described:

COF=Torque13×∅×nF

Two commercial VS composed of crosslinked HA biomaterials references (NASHA, Hylan G-F 20*)* were used as comparators to KiOmedine^®^ CM-Chitosan. The polymer-free isosmotic buffer of KiOmedine^®^ CM-Chitosan and synovial fluid from osteo-arthritic (OASF) patients were used as negative controls. OASF was obtained by an orthopaedic surgeon from patients who were suffering from knee OA and undergoing total knee replacement (ethics committee agreement of Catholic Universit of Louvain n° B403201111664). A total of ten (10) OASF samples were collected from patients aged 60–77 years at the time of surgery. Synovial fluid was collected in lithium-heparin tubes (Sarstedt, Nümbrecht, Germany) and stored frozen at -20°C until analysis.

#### *Ex-vivo* biomechanical kinematic study

An *ex-vivo* ovine model of 3D joint mobility was set up for the biomechanical characterization of KiOmedine^®^ CM-Chitosan and Hylan G-F 20 formulations. The Buffer of KiOmedine^®^ CM-Chitosan was used as a negative control.

Immediately after euthanasia (intravenous administration of 150mg/kg of pentobarbial), the hindlimbs from five healthy adult ewes (Ile de France, aged 3 to 7 years, and weighing 70 to 80 kg) were detached at the coxo-femoral joint. The experimental protocol (KI 10/148) was approved by the local ethical committee of university of Namur for animal welfare (ethics committee agreement n° UN PM 19/06 VA). This sample size was suitable for parametric statistics.

The skin was removed and thigh muscles were transected, with the exception of the distal insertion of the quadriceps femoris on the patella. The joint capsule was carefully preserved. The dissections were performed by a qualified veterinary surgeon. The femur was preserved and its proximal end fixed in a resin cube to enable adequate positioning of the bone in the biomechanical testing device. The limb was moistened, wrapped in gauze, sealed in plastic bags, and stored frozen until 24 hours before the test. The limbs were thawed for 6 hours at room temperature and stored at 2–8°C until testing.

The biomechanical kinematic testing device consisted of a high-precision tensile test machine (LSi) that was able to apply a predefined load on clamped hindlimbs and a stereo-photogrammetric system that was composed of 10 cameras (Optitrack) and was used to record the kinematics of the knee joint during the investigated activity.

On the day of the experiment, optical custom-made reference frames were rigidly attached to the proximo-medial aspect of the tibia and on the disto-medial aspect of the femur of each dissected limb. In each frame, several spheres for reflecting infrared light were mounted in order to enable the 3D motion tracking of each limb in the space by the motion analysis system.

**Fig 1:**
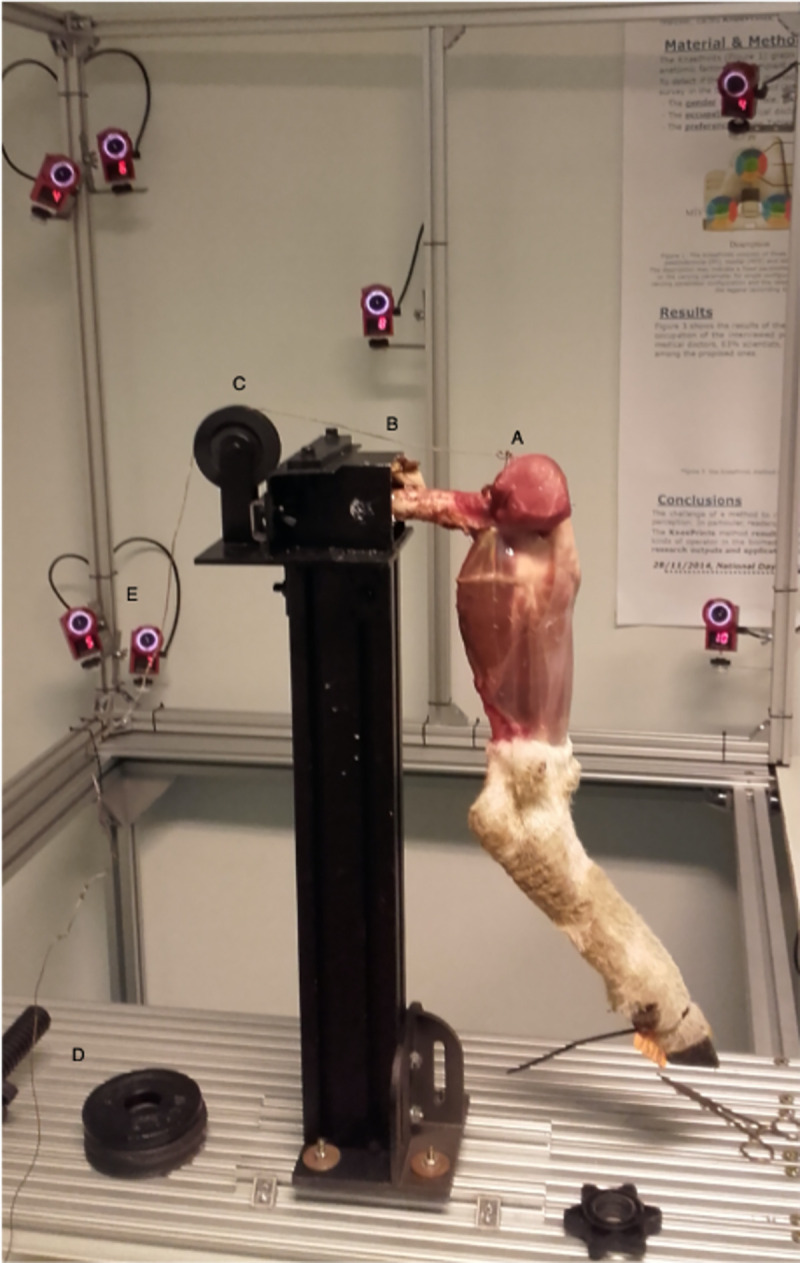
Biomechanical *ex vivo* sheep model of 3D joint mobility. The distal portion of the gastrocnemius and its tendon are tightened by a Colson ring (A). A metallic wire (B) is connected, via a pulley (C), to a metallic bar to which weight plates are loaded (D). The stereo-photogrammetric system (Optitrack) is composed of 10 cameras and 2 optical references frames (rigidly attached to the tibia and the femur) to record and measure the kinematics of a knee joint (E).

The limb, via the resin cube, was fixed horizontally on a metal frame of the biomechanical testing device (Fig 1).

**Fig 2:**
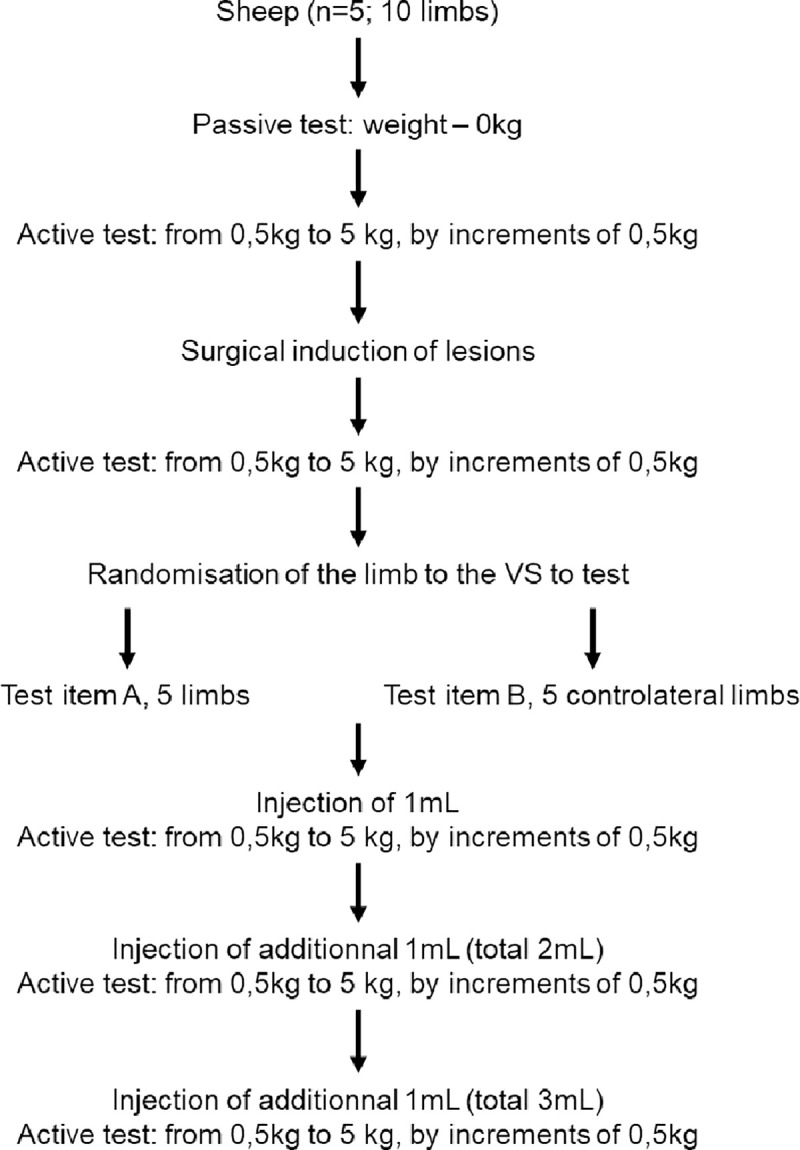
Analytical set-up of the *ex-vivo* biomechanical study. The experimenter pulled the wire manually (passive test) to check the dispositive. Then the quadriceps was loaded with an initial weight of 0.5 kg and the motion of 3 flexion-extension cycles of the leg was recorded with increasing weight for up to 5 kg by 0.5 kg increment. Test item A = Hylan G-F 20 and test item B = KiOmedine® CM- chitosan.

The distal limb was allowed to move freely. The remaining portion of the quadriceps femoris was tightly fixed via a Colson ring. A stainless-steel wire, via a pulley, connected the ring to a metallic bar on which weight plate were loaded. Flexion-extension cycles of the leg were recorded according the protocol descried in Fig 2.

**Fig 3:**
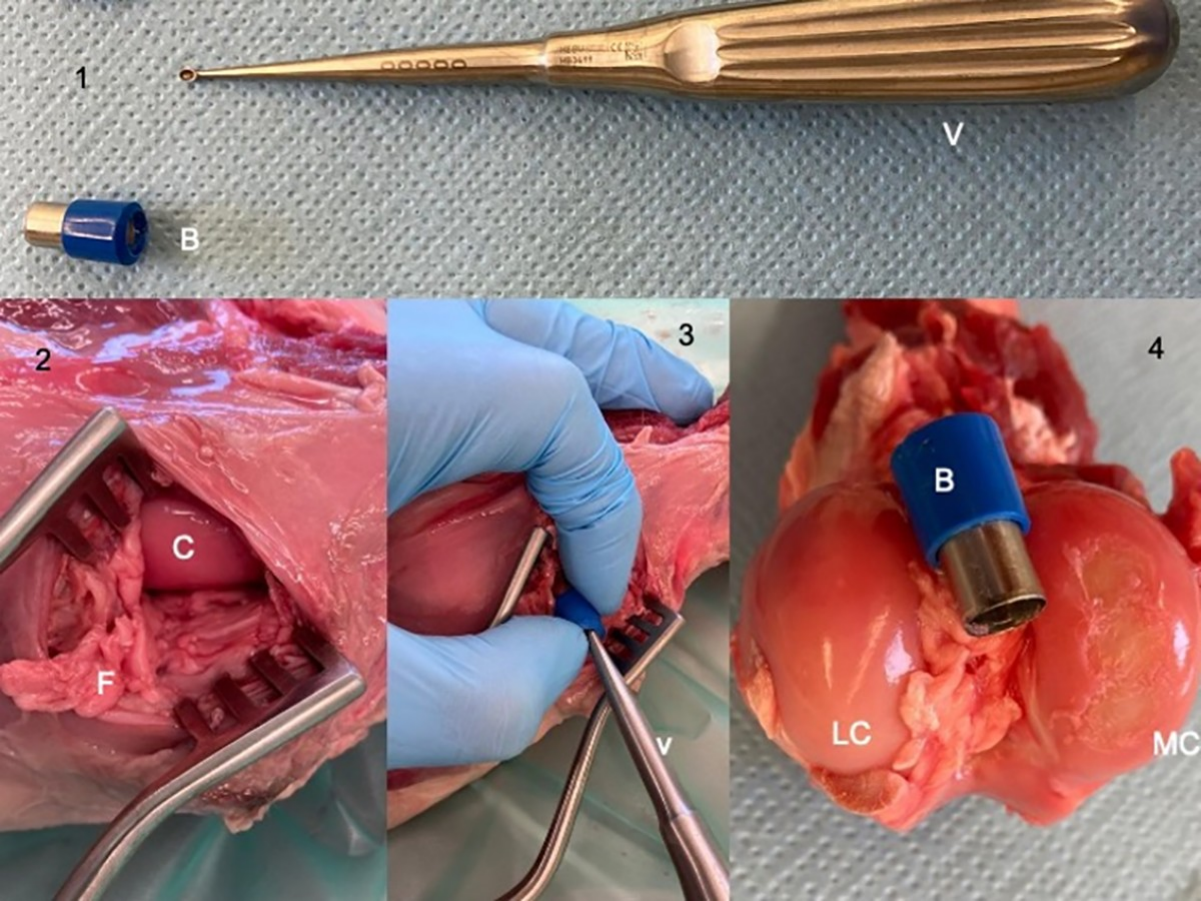
Procedure of creation of cartilage default. (1) The distal and cutting part was sawed from a biopsy punch (B). (2) After incision of the subcutis and joint capsule, the medial condyle (C) of the femur was exposed. Fat (F) was removed from the joint cavity. (3) The biopsy punch (B) was deeply entered into three adjacent sites of cartilage in the axial aspect of the medial condyle of the femur. The Volkman curette (V) was used to remove cartilage. Then the joint capsule was sutured and tests were performed. (4) Picture 4 illustrates the three cartilage defects on the medial condyle (MC) at gross anatomy dissection. LC = lateral condyle.

In order to create standardized cartilage changes, a cartilage defect was created by a surgical technique. The axial aspect of the medial femoral condyle was chosen as the site for the induction of lesions since a prevalence study demonstrated that it was a site of naturally occurring cartilage defects in this population of research sheep [31]. To create chondral lesions on both (the left and right) limbs, the joint capsule was minimally incised on 5 cm medially to the patellar ligament, with the leg in flexion. Fat was removed to expose the medial condyle. Three 8 mm diameter chondral defects were created in situ by drilling the cartilage deep to the calcified layer using an 8 mm diameter biopsy punch to delineate the lesion and a Volkmann curette to remove the cartilage (Fig 3). The articular capsule was then closed with vicryl 2.0 in a continuous pattern. Flexion-extension cycles of the leg were recorded again (with increasing weight for up to 5 kg by 0.5 kg increment) after induction of the cartilage defects. The left and right limbs of each sheep were then randomly allocated to the injection of one VS (KiOmedine^®^ CM-Chitosan or Hylan G-F 20) and the controlateral limbs received the second VS (Hylan G-F 20 or KiOmedine^®^ CM-Chitosan). Flexion-extension cycles were repeated after every IA injection of increasing volumes of VS (1 mL to 3 mL) (Fig 2).

**Fig 4:**
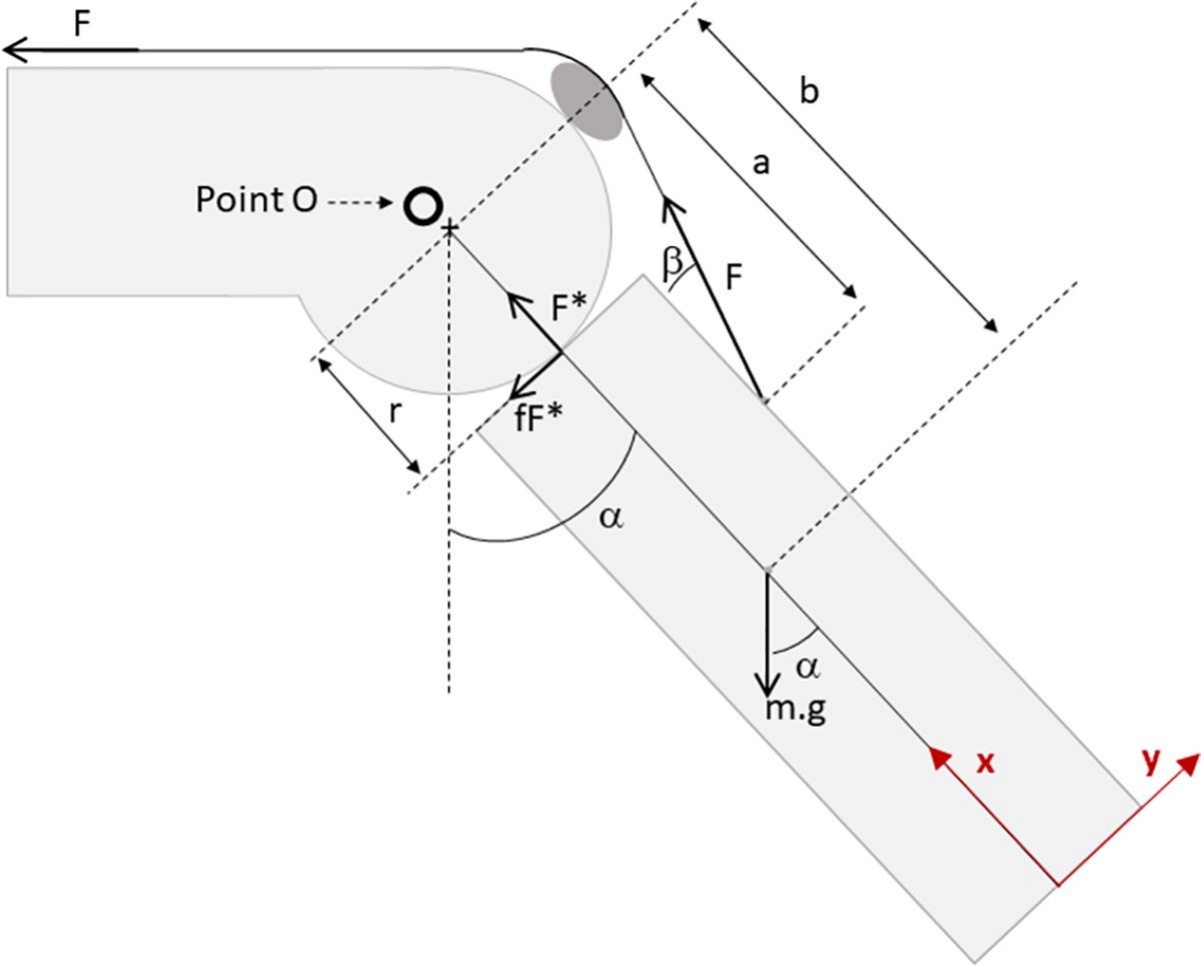
Schematic representation of force involved during the ex-vivo model. Calculations were performed, with the assumption that the ovine hindlimb is a 2D hinge joint that is rotating around the O point, due to the quadriceps force (F), involving the following parameters:
F = quadriceps force (in the experimental analysis obtained as the force of the weight);F_x_ = component of the force F along the direction XF_y_ = component of the force F along the direction Ym = mass of the limb;mg = weight of the limb (g is the gravity, 9.81 m/s^2^);F* = the contact force between the tibia and the femur;f = friction coefficient (or COF);fF* = the friction force;r = radius of the femoral condyle;a = distance between the tibial insertion point of the patellar tendon and the hinge centre;b = distance between the barycentre of the lower leg and the hinge centre;α (alpha) = flexion angle;$\ddot{\alpha }$ = the second derivative (angular acceleration) of α with respect to the timeI_0_ = the mass moment of inertiaβ (beta) = angle from the patellar tendon and the tibial axis.

Knee joint kinematics were analyzed in terms of flexion and extension, internal and external rotation, and cranio-caudal translation of the femur with respect to the tibia, according to the schematic representation of the ex-model described in Fig 4. The cartilage-cartilage COF was determined based on knee joint kinematics. Parameters in damaged cartilage conditions were compared to those in undamaged condition in order to estimate the recovery of joint motion after a VS injection. They were also compared between KiOmedine^®^ CM-Chitosan or Hylan G-F 20.

The equation of dynamics in the case of pure rotation enables the calculation of the missing information, i.e. F*, f (COF) and the beta angle, according to Eqs 1 to 5 [38].

Eqs 1 to 3:

The sum of all the force in the direction X are null (it is considered a pure rotation and therefore no translation): ∑*F_x_* = 0

$$F^{*}=mg\;\cos\;\alpha -F\;cos\;(\beta )$$ (1)

The sum of all the force in the direction Y are null (it is considered a pure rotation and therefore no translation): ∑*F_y_* = 0

$$f=\frac{F\;\sin\;\beta -mg\;\sin\;\alpha }{mg\;\cos\;\alpha -F\;\cos\;\beta }$$ (2)

Equation is the equation of rotation: The sum of all the torque (M) provide a certain rotation that is proportional of the Inertia moment on the rotation axis: $\sum M=I\ddot{\alpha }\to$

$$\sin\;\beta =\frac{I_{O}\ddot{\alpha }+mg(b-r)\sin\;\alpha }{F(a-r)}$$ (3)

For each configuration, i.e. healthy (H), with a lesion (L) and treated by injection of the test product (T), and for the different weights applied (W), the range of motion (ROM) was measured as in Eq 4:

$$ROM={\left(Max\;\alpha -Min\;\alpha \right)}_{W}$$ (4)

For the L and T conditions, considering n as the number of weight repetition used for the test, the mobility (MOB) is defined as in Eqs 5 and 6:

$${MOB}_{L}=100-\frac{1}{n}\sum_{n}{\left(\frac{{ROM}_{L}-{ROM}_{H}}{{ROM}_{H}}\right)}_{W}$$ (5)

$${MOB}_{T}=100-\frac{1}{n}\sum_{n}{\left(\frac{{ROM}_{T}-{ROM}_{H}}{{ROM}_{H}}\right)}_{W}$$ (6)

The “recovery of joint motion” (REC) thanks to the treatment with the test product is calculated via Eq 7:

$$REC=\frac{{MOB}_{T}-{MOB}_{L}}{{MOB}_{H}-{MOB}_{L}}*100$$ (7)

After the procedures, gross anatomy was performed. After careful dissection, the cartilage was kept moist by covering the joint surface with gauze sponges soaked in lactated Ringer’s solution. The distal articular surface of the femur, proximal articular surface of the tibia and articular surface of the patella were examined by gross observation. Synovial membrane was observed as well. OARSI (Osteoarthritis Research Society International) recommendations for macroscopic scoring of cartilage and synovium in sheep were used. Gross observations of cartilage and synovium were compared to articular changes that are expected to occur naturally in a population of healthy (without signs of OA) research sheep [31] and demonstrate that limbs were normal at the start of the study. Left and right limbs were compared to demonstrate that scoring was similar on both limbs, and that defects that had been created were of similar size and similar location (Fig 3).

### Free radical scavenging activity

#### Trolox equivalent antioxidant capacity (TEAC) assay

The free radical scavenging capacity of KiOmedine^®^ CM-Chitosan, Hylan G-F 20 and NASHA formulations was evaluated using the *in-vitro* trolox equivalent antioxidant capacity (TEAC) assay as previously described [39].

ABTS (2,2′-azinobis(3-ethylbenzothiazoline-6-sulfonic acid or Trolox)) radical cation ABTS֯^+^, was produced by reacting 7 mM ABTS stock solution with 2.45 mM potassium persulfate. The mixture was placed in the dark at room temperature for 12–16 hours before use. From there, the ABTS֯^+^ solution was diluted in water and mixed with the sample to a final absorbance of 0.6. Each sample was tested at a final concentration of polymer of 0.8% in triplicate. The polymer-free isosmotic buffer of KiOmedine^®^ CM-Chitosan was used as the negative control and 3 mM ascorbic acid as the positive control. Free radical scavenging capacity was reported in the Trolox equivalent.

### Statistical analyses

Descriptive statistical analyses were performed using mean, median, standard deviation (SD), and minimum and maximum values for continuous variables. Inferential statistical analyses included ANOVA or t-test and were performed with a significance level of 5% and 90% power assuming normality. Where appropriate, Bonferroni’s correction for multiplicity was applied for multiple comparisons.

## Results

### Lubrication capacity

**Fig 5:**
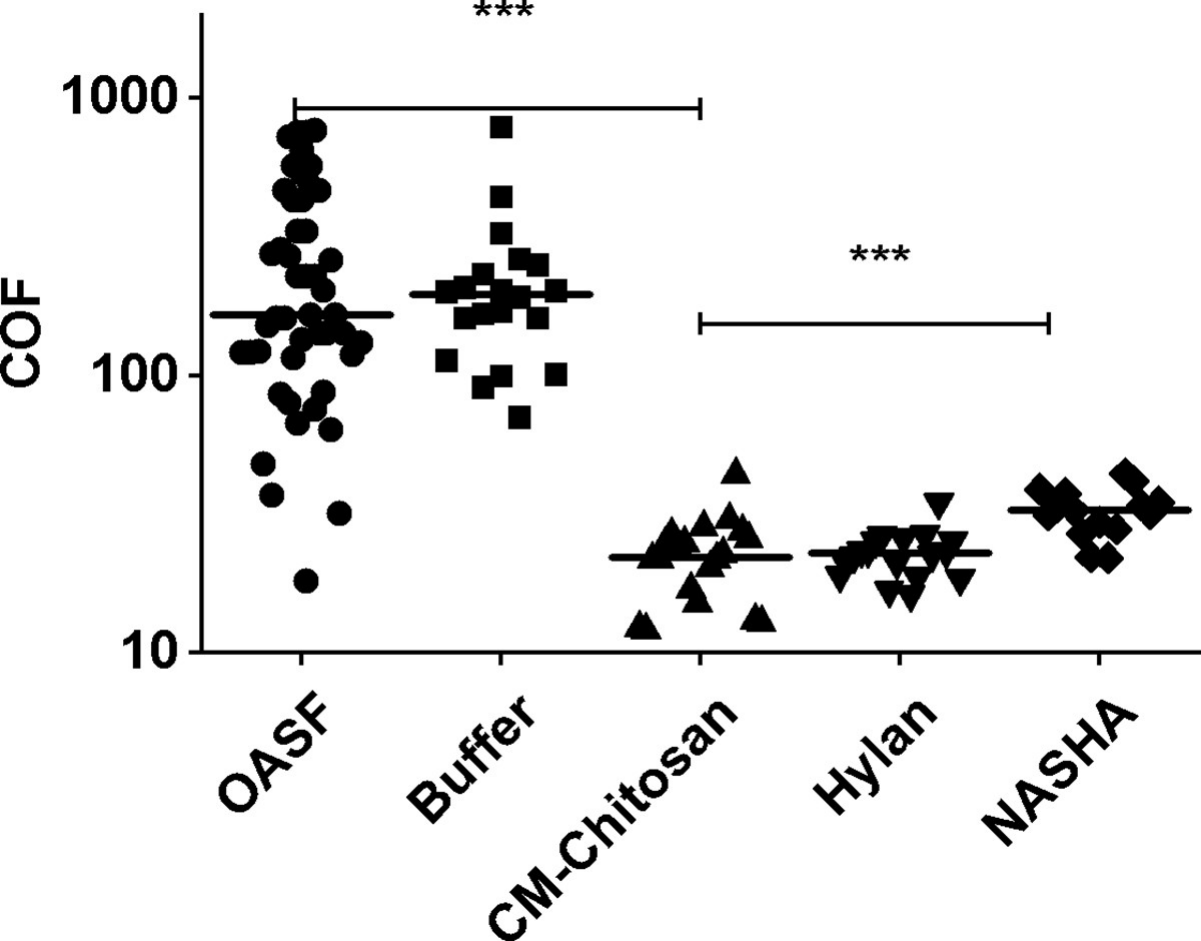
COF measurements of KiOmedine® in the p-HEMA tribological model. The lubrication capacity of KiOmedine® (N = 4) to reduce COF between the disks in rotating friction was compared to two commercial VSs biomaterial references composed of crosslinked hyaluronic acid, Hylan G-F 20 (N = 4) and NASHA(N = 3), the buffer control (N = 4) and synovial fluids punctured from osteoarthritic patients (N = 10). KiOmedine® exhibited similar or higher lubricating capacity compared to Hylan G-F 20 and NASHA (ANOVA, ***p < 0.0001).

Based on tribological characterization, KiOmedine^®^ CM-Chitosan showed a high lubrication capacity (Fig 5) as evidenced by a COF reduction (22±8, mean±SD) when compared to the buffer and OASF (222±159 and 269±217, respectively). No significant difference in COF was reported between KiOmedine^®^ CM-Chitosan and Hylan G-F 20 (23±5). However, when compared to KiOmedine^®^ CM-Chitosan, NASHA had a significantly higher COF (33±6).

**Fig 6:**
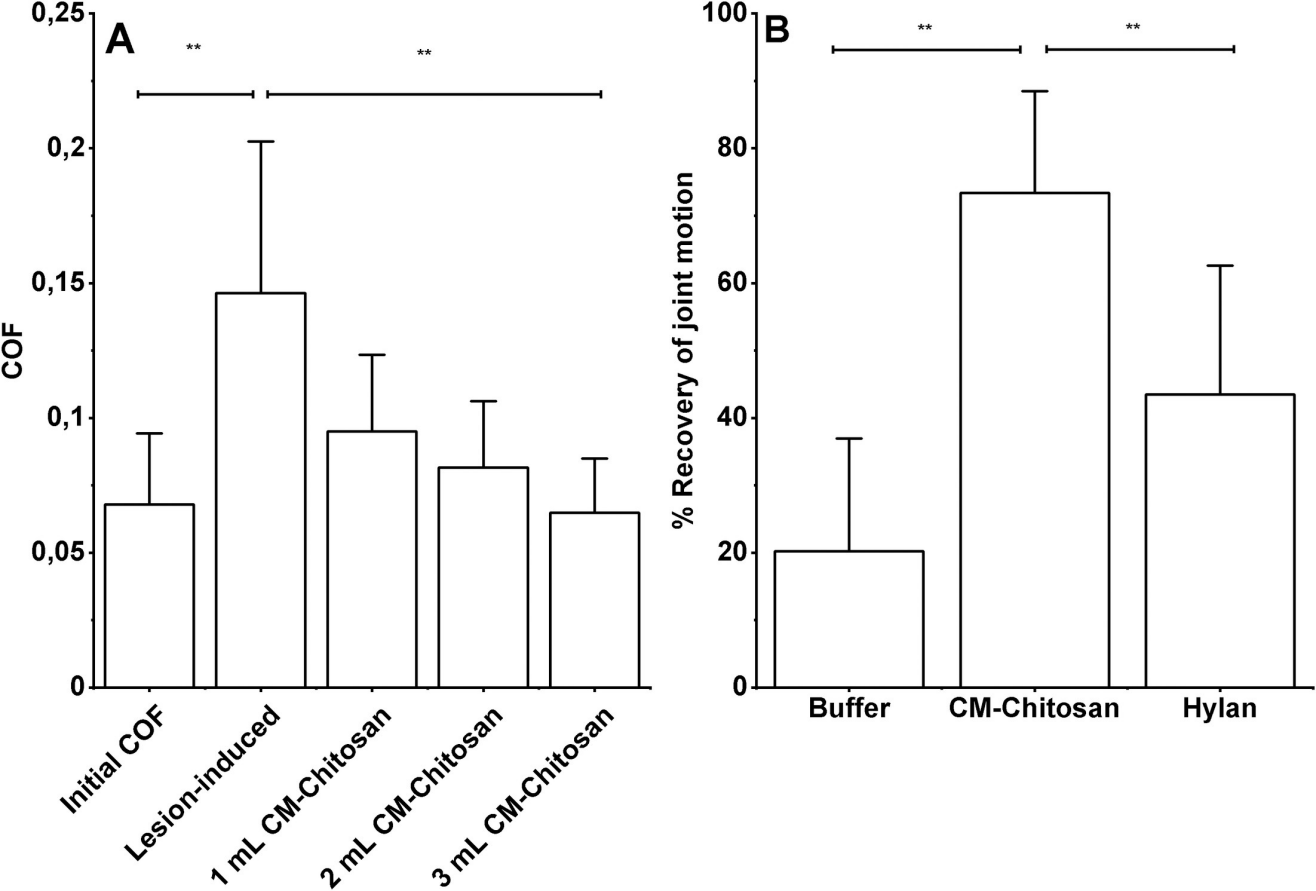
Lubrication capacity of KiOmedine® CM-chitosan in the biomechanical *ex-vivo* sheep model of 3D joint mobility. (A) A significant increase in COF is observed due to increased joint friction in damaged cartilage (paired T-test, **p<0.01); COF is then progressively significantly improved following the IA injection with KiOmedine® CM-chitosan in a volume-dependent manner (ANOVA, **p<0.01). (B) KiOmedine® CM-chitosan was compared to Hylan G-F 20 at 3 ml IA volume and showed significantly better recovery of joint motion loss on damaged cartilage (t-test, **p<0.01); the effect of the polymer-free buffer of KiOmedine® on the recovery of motion was low.

To distinguish the lubrication capacity of KiOmedine^®^ CM-Chitosan and Hylan G-F 20, these formulations have been compared in the *ex-vivo* biomechanical kinematic model. Gross anatomy performed after the test demonstrated that joints were normal in all anatomical sites in all animals. The created defects were similar in both the left and right limb of every animal. COF was significantly increased after the induction of defects versus the initial non-operated condition (Fig 6A), leading to a reduction in joint motion of 16±10% (mean±SD). Following the IA injection in the injured knee, the lubrication effect of KiOmedine^®^ CM-Chitosan was correlated with a decrease of COF in a volume-dependent manner. The recovery of joint motion was estimated at 3 ml of KiOmedine^®^ CM-Chitosan, and that volume was used to compare KiOmedine^®^ CM-Chitosan and Hylan G-F 20. A significant recovery of joint motion was observed with KiOmedine^®^ CM-Chitosan (73 ± 15%), which was pointedly superior to the recovery capacity of Hylan G-F 20 at the same injection volume (43 ± 19%) (Fig 6B). The effect of the polymer-free buffer of KiOmedine^®^ on recovery of motion was low confirming that the lubrication capacity of KiOmedine^®^ was attributable to CM-Chitosan.

### Free radical scavenging activity

**Fig 7:**
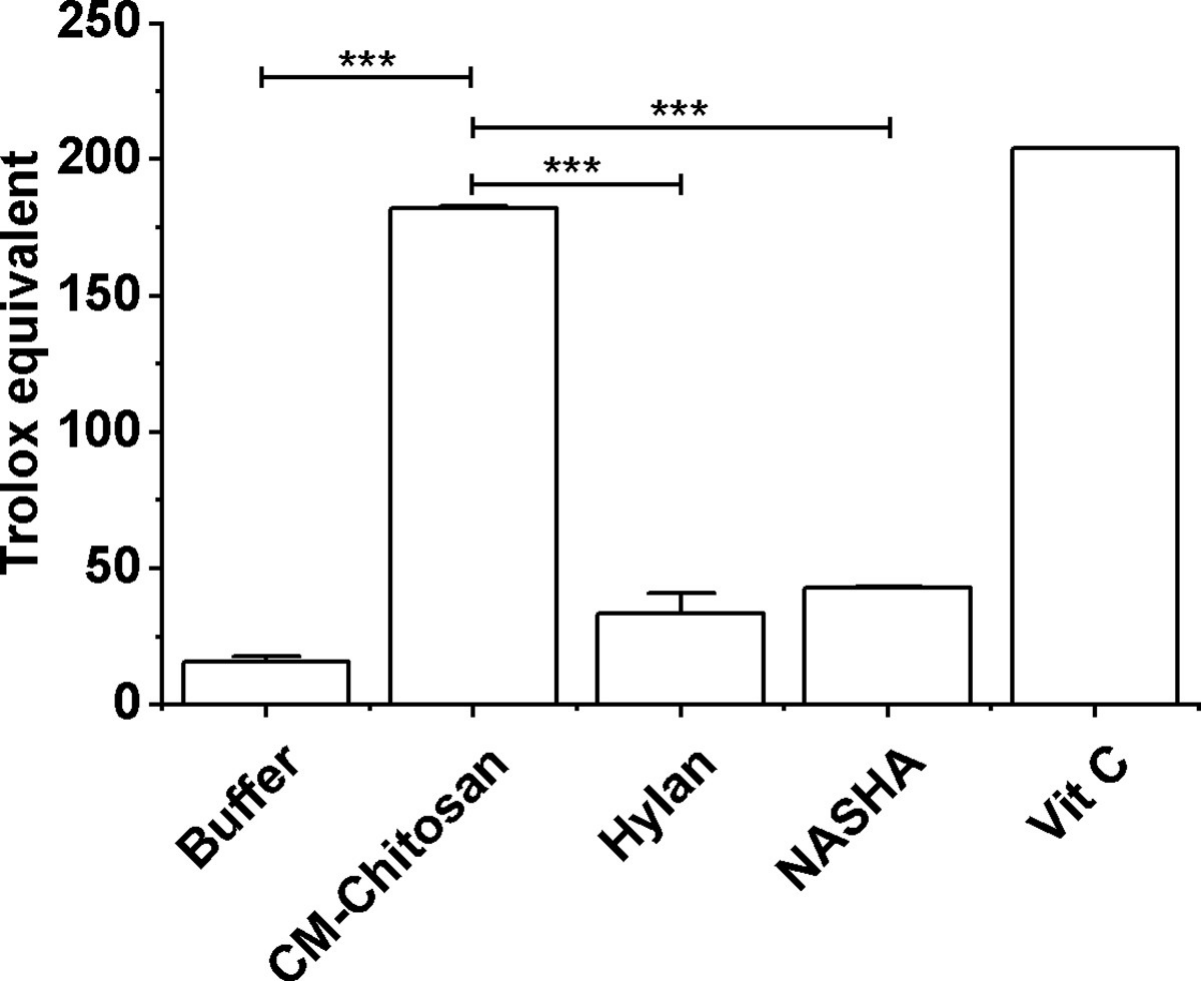
Free radical scavenging capacity using the TEAC assay. KiOmedine® CM-chitosan was compared to Hylan G-F 20, NASHA, negative and positive controls respectively, the polymer-free buffer of KiOmedine® CM-chitosan and vitamin C. KiOmedine® CM-chitosan showed high free radical scavenging capacity *in vitro* as compared to crosslinked HA biomaterials, Hylan G-F 20, and NASHA (ANOVA, ***p<0.0001).

KiOmedine^®^ CM-Chitosan showed high free radical scavenging capacity in the TEAC assay (182±1), which was significantly superior when compared to the crosslinked HA-based biomaterials Hylan G-F 20 (33±4) and NASHA (43±0) (Fig 7). Moreover, the buffer used in CM-Chitosan formulation had no scavenging activity (16±2).

## Discussion

In the knee joints of patients suffering from OA, SF was characterized by its loss of lubrication capacity inducing an increased friction between cartilage-cartilage surfaces. High COF (269±217) was measured during the tribological characterization of OASF from 10 patients in the current study. Because of differences in progress of OA in patients, this loss of lubrication capacity could be more or less pronounced and it explained the variability of COF measurement observed in this *in-vitro* model. The reduction of rheological property of the SF (i.e. the loss of lubrication capacity) was likely due to the decrease of the mean molecular weight and concentration of HA in SF. One possible cause of this is the oxidative degradation of endogenous HA by free radicals. VS injection is a common procedure to reduce the OA symptoms. The first role of VS is to support the lubrication capacity of the synovial fluid [40].

KiOmedine® CM-Chitosan has demonstrated lubrication capacity in the two complementary models in the current study. This property was directly attributed to the presence of CM-Chitosan used in KiOmedine^®^ when compared to its polymer-free buffer. Moreover, in the *in-vitro* study, the lubricating capacity of KiOmedine^®^ CM-Chitosan was similar to Hylan G-F 20, and better than NASHA. In the *ex-vivo* model, the recovery in motion brought by the injection of KiOmedine^®^ CM-Chitosan was significantly better than that brought by the same volume (3 mL) of Hylan G-F 20.

Lubrication is a process that aims at reducing friction between two moving pieces. There are three different types of lubrication: boundary, mixed, and full film. Joint lubrication is ensured via a combination of regimes. Spreading of the SF into a film of gradually diminishing thickness in the direction of sliding generates a pressure that balances the applied load. This regime mechanism relies on viscosity, the thickness, and the shape of the SF film, and the speed of the relative movement of the articular surfaces. When the load increases and surface separation decreases, the regime changes from film lubrication to boundary lubrication.

Boundary lubrication is defined as that in which the sliding surfaces are separated by a very thin molecular film of lubricant, which means that the chemical and physical natures of the surfaces and the lubricant are of major importance.

A single monolayer of SF molecule components is adsorbed on the cartilage surface preventing direct surface-to-surface contact and protecting cartilage from excessive wear and friction [41]. The *in-vitro* tribological model allows one to study the boundary–lubricant capacity. Chitosan [42] and HA [43] can interact with p-HEMA based surfaces that form end-grafted polymer brush-like structures and contribute to boundary lubrication mechanism. The term polymer brush refers to a class of thin polymer coatings in which each of the chains is tethered to an underlying substrate. Both polymer surfaces are charged, which allows them to strongly bind to water molecules, entrapping them between the brush-like polymer chains. On the boundary regime, loads increase and surfaces are compressed, polymer chains are flattened, and the trapped water molecules form a hydrated layer that can support the load and reduce shear forces.

VSs based on highly crosslinked HA, such as NASHA, either do not have or have insufficient available polymer chains to form brush-like structures and their capacity to reduce friction in boundary lubrication is therefore limited. On the other hand, KiOmedine^®^ CM-Chitosan, which is composed of non-crosslinked CM-Chitosan, and Hylan G-F 20, which contains 80% of linear HA, have sufficient linear polymer chains to available coat p-HEMA surfaces. This most likely explains why in the tribological *in-vitro* model the lubricating capacity of KiOmedine^®^ CM-Chitosan was found to be similar to Hylan G-F 20 and better when compared to NASHA.

However, this tribological model has some limitations as it does not reproduce the biochemical composition of cartilage, cartilage-on-cartilage surface contact, and a joint sliding motion.

For this reason, the lubrication capacity of KiOmedine^®^ CM-Chitosan and Hylan G-F 20 was then studied in a newly developed *ex-vivo* biomechanical model. This model used real cartilage surfaces and reproduced a realistic cartilage-on-cartilage movements that operates without 3D constraints which could impact the natural movements [44].

It was observed that joint motion increased proportionally to the injected volume of KiOmedine^®^ CM-Chitosan. For the same volume injected, the recovery in motion was significantly better for KiOmedine^®^ CM-Chitosan than for Hylan G-F 20. This superior lubricating capacity of KiOmedine^®^ CM-Chitosan observed in this biomechanical model could be explained by the presence of residual mucin-like glycoproteins. Mucin-like glycoproteins, such as lubricin, are present in the superficial layer of articular cartilage [45]. These proteins can bear hydrophilic hydroxyl, carboxyl, sulphate, and amino groups, and form lubricating gels through intermolecular complexation [46, 47]. Mucin-like glycoproteins function as effective boundary lubricants in low viscosity conditions [48], forming thin gel films [49] that repel interfacial contact. In this biomechanical *ex-vivo* model, CM-Chitosan and HA chains could likely interact with lubricin that is present in the cartilage surface forming mucin-like glycoproteins structures to create a brush-like boundary lubrication layer onto the cartilage surface. Compared to Hylan G-F 20 at an equivalent injected volume, CM-Chitosan has a higher availability of non-crosslinked polymer chains and functional groups to form complex structures with lubricin and then to create a brush-like boundary lubrication layer.

In this study, the two tribological models, *in-vitro* and *ex-vivo*, provided different results. The *in-vitro* model revealed similar lubrication capacity of KiOmedine® CM-Chitosan and Hylan G-F 20, while the *ex-vivo* model underlined superior lubricating capacity of KiOmedine® CM-Chitosan. The contact geometry and the nature of the counter-surface have a significant impact on the experimentally determined friction force. The compliance of the interacting surfaces was also different. Thus, the results obtained by the two models may not be directly comparable.

Of course, the ex vivo model developed and used in the current study is not a model of OA, since the lesions are not the result of a disease such as when it is induced by surgery. We cannot therefore conclude that the effects of the tested products would be the same in vivo in natural or induced OA. It is actually a model of chondral defects with the advantage that lesions are of similar size and locations in all limbs.

Another action to relieve OA symptoms is to slow down the oxidative degradation of synovial components. In the TEAC model, KiOmedine® CM-Chitosan has demonstrated free radical scavenging capacity. This property was directly attributed to the presence of CM-Chitosan used in KiOmedine^®^ when compared to its polymer-free buffer. Moreover, KiOmedine® CM-Chitosan had a higher ability to scavenge free radicals compared to crosslinked HA formulations. Due to this capacity, the KiOmedine® CM-Chitosan is able to delay HA degradation in an oxidative environment [50]. This effect was attributed to the polymer itself, as the buffer had no scavenging activity even when supplemented with sorbitol.

The free radical scavenging capacity of carboxymethyl chitosan is generally recognized, but its mechanism is not clearly elucidated [51–53]. One hypothesis for this is the reaction of free radicals with active hydroxyl or amine functions present on the backbone of chitosan and its derivatives to form stable macromolecules [54, 55]. HA is also known to have antioxidant properties but the mechanism is also not clearly understood, even if we can consider that the available hydroxyl functions present on the backbone can have, as is the case for chitosan and its derivatives, a positive effect by trapping free radicals, such as ROS. It was demonstrated that the lowest molecular weight HAs had the highest antioxidant activity [56]. In crosslinked HA hydrogel, the crosslinking reaction induces a steric hindering; the functions of the polymer backbone known to participate in the capture of free radicals are therefore less accessible. This could explain the lower free radical scavenging capacity of these crosslinked HA formulations. One limitation of this study is the radical used in TEAC assay. Even if this test is well-known [57] to assess and compare free radical scavenging capacity of different product, the ABTS radical is not specific of an *in-vivo* environment.

## Conclusion

In this study, KiOmedine® CM-Chitosan demonstrated two essential properties that are sought to ensure viscosupplementation of joints. The capacity to provide cartilage-cartilage lubrication and improve joint motion was tested in two innovative tribological models. Further studies will be designed to determine the interaction of the KiOmedine®CM-chitosan with constituents of synovial fluid such as lubricin, to reinforce our comprehension of its mechanism of action in the treatment of OA symptoms. KiOmedine®CM-chitosanIt has also shown a good ability to scavenge free radical. This capacity could protect synovial components like endogenous HA from oxidative degradation. However, the mechanism is not clearly elucidated. In next works, the impact of chemical structure of KiOmedine® CM-chitosan radical scavenging capacity will be further investigated on different reactive oxygen species.

KiOmedine® CM-Chitosan has demonstrated its superiority over crosslinked HA formulations in terms of free radical scavenging capacity and it appears that KiOmedine® CM-Chitosan seems to have a higher lubrication capacity. This difference could be explained by the differences in polymer chemical structure but also by how they were formulated. Contrary to cross-linked HA, KiOmedine® CM-Chitosan has amine functions in addition of hydroxyl functions. Moreover KiOmedine® CM-Chitosan is a linear polymer with higher chain mobility than crosslinked HA, which probably enables easier interactions between CM-Chitosan and the joint tissue. In addition to these non clinical performance tests, a recent clinical study (clinical trial APROOVE NCT03679208 (www.clinicaltrials.gov)) was conducted and demonstrated the safety and 6-months performance of KiOmedine® CM-Chitosan to reduce OA symptoms after a single (3mL) injection in OA patients.

## Supporting information

S1 Data COF (Fig 6A)—Raw data + data for graph representation (green highlighted) of Fig 6A. (PDF)

S2 Data COF measurements (Fig 5)—Raw data of Fig 5. (PDF)

S3 Data COF measurements (Fig 5)—Box plot for representation of Fig 5. (PDF)

S4 Data Data free radical scavenging capacity (Fig 7)—Raw data + data for graph representation of Fig 7. (PDF)

S5 Data Data ROM (Fig 6B)—Raw data + data for graph representation (green highlighted) of Fig 6B. (PDF)
