# Supplementary material for: Non-clinical assessment of lubrication and free radical scavenging of an innovative non-animal carboxymethyl chitosan biomaterial for viscosupplementation: An in-vitro and ex-vivo study
Source: PLoS One. 2021 Oct 11;16(10):e0256770. doi: 10.1371/journal.pone.0256770 (PMC8504732; doi:10.1371/journal.pone.0256770)
Supplement: S1 Data — (PDF) [file pone.0256770.s001.pdf]

	CoF Limb 1	CoF Limb 2	CoF Limb 3	CoF Limb 4	CoF Limb 5	**Mean**	**SD**
initial COF	0,0971	0,0953	0,056	0,0498	0,041	**0,06784**	**0,02644**
Lesion-induced	0,2145	0,1976	0,12558	0,10296	0,091	**0,14633**	**0,05623**
1 mL CM-Chitosan	0,12727	0,1196	0,0923	0,0754	0,06032	**0,09498**	**0,02846**
2 mL CM-Chitosan	0,1074	0,108	0,0732	0,0648	0,05472	**0,08162**	**0,02469**
3 mL CM-Chitosan	0,0894	0,082	0,059	0,0513	0,0426	**0,06486**	**0,02006**
