# Supplementary material for: Non-clinical assessment of lubrication and free radical scavenging of an innovative non-animal carboxymethyl chitosan biomaterial for viscosupplementation: An in-vitro and ex-vivo study
Source: PLoS One. 2021 Oct 11;16(10):e0256770. doi: 10.1371/journal.pone.0256770 (PMC8504732; doi:10.1371/journal.pone.0256770)
Supplement: S2 Data — (PDF) [file pone.0256770.s002.pdf]

OASF	Buffer	CM-Chitosa	Hylan	NASHA
535,23097	99,4609	28,2809	25,84052	29,48725
134,88245	169,92608	15,10464	21,13416	22,12474
228,16374	90,60784	20,24747	16,11432	21,96895
115,18649	203,24302	16,81563	16,69661	26,84653
165,47125	166,7259	21,60679	18,81021	27,92245
429,35687	789,00857	24,86413	26,28483	32,58896
753,50294	232,08416	22,69488	26,56626	44,17057
227,052	262,66775	23,48228	25,13473	37,48293
160,53008	441,4275	30,22538	22,44893	41,53276
141,55649	191,62416	26,7187	23,34999	35,08526
429,35687	207,76161	43,78432	34,75024	33,94381
753,50294	325,23815	24,58644	23,38164	31,3788
227,052	250,2616	27,26655	23,14652	31,73437
160,53008	160,63837	21,64146	22,23023	38,77943
141,55649	160,91233	25,63126	25,31107	34,87353
270,51599	113,05938	21,61551	20,87228	
202,53198	200,38854	13,08732	18,4432	
283,72338	100,59493	12,01216	18,95538	
260,13364	202,20261	12,86279		
274,71898	70,64761	12,24477		
767,08629				
643,57668				
568,95118				
482,7797				
724,3973				
17,99908				
150,43445				
164,42526				
465,73306				
568,61913				
464,70882				
329,86756				
331,09532				
121,71806				
66,73112				
75,07638				
79,44483				
87,13805				
141,74741				
120,78422				
118,33858				
84,8988				
63,73331				
121,02001				
130,97966				
36,96673				
31,78123				
47,70199				
