# Supplementary material for: Non-clinical assessment of lubrication and free radical scavenging of an innovative non-animal carboxymethyl chitosan biomaterial for viscosupplementation: An in-vitro and ex-vivo study
Source: PLoS One. 2021 Oct 11;16(10):e0256770. doi: 10.1371/journal.pone.0256770 (PMC8504732; doi:10.1371/journal.pone.0256770)
Supplement: S3 Data — (PDF) [file pone.0256770.s003.pdf]

Statistics on Columns (02-07-21 15:02:22)

Descriptive Statistics

	Mean	Median	P25	P75
OASF	268,79771	164,94826	119,5614	429,35687
Buffer	221,92405	196,00635	136,84888	241,17288
CM-Chitosan	22,23867	22,16817	15,96014	26,17498
Hylan	22,7484	22,79773	18,95538	25,31107
NASHA	32,66136	32,58896	27,92245	37,48293
