# Supplementary material for: Non-clinical assessment of lubrication and free radical scavenging of an innovative non-animal carboxymethyl chitosan biomaterial for viscosupplementation: An in-vitro and ex-vivo study
Source: PLoS One. 2021 Oct 11;16(10):e0256770. doi: 10.1371/journal.pone.0256770 (PMC8504732; doi:10.1371/journal.pone.0256770)
Supplement: S4 Data — (PDF) [file pone.0256770.s004.pdf]

	Raw data Trolox Eq	Graph	
		Mean	SD
CM-Chitosan	181,32<br>182,21<br>182,89	182,14	0,79
HYLAN	37,46<br>24,95<br>38,08	33,50	7,41
NASHA	43,68<br>42,95<br>43,37	43,33	0,37
Vit C	204,37<br>204,21	204,29	0,11
Buffer	13,61<br>24,48<br>10,76	16,28	7,24
