# Supplementary material for: Non-clinical assessment of lubrication and free radical scavenging of an innovative non-animal carboxymethyl chitosan biomaterial for viscosupplementation: An in-vitro and ex-vivo study
Source: PLoS One. 2021 Oct 11;16(10):e0256770. doi: 10.1371/journal.pone.0256770 (PMC8504732; doi:10.1371/journal.pone.0256770)
Supplement: S5 Data — (PDF) [file pone.0256770.s005.pdf]

	ROM Limb 1	ROM Limb 2	ROM Limb 3	ROM Limb 4	ROM Limb 5	**Average**	**SD**
Buffer	43,09574	30,22475	4,76514	4,11709	18,7659	**20,19372**	**16,76053**
CM-Chitosan	75,17042	65,76376	52,10494	90,68065	83,25105	**73,39416**	**15,08799**
Hylan	39,84012	50,46091	12,92601	50,02492	64,09178	**43,46875**	**19,12596**
